# Determinants of arsenic methylation efficiency and urinary arsenic level in pregnant women in Bangladesh

**DOI:** 10.1186/s12940-019-0530-2

**Published:** 2019-11-05

**Authors:** Shangzhi Gao, Pi-I Lin, Golam Mostofa, Quazi Quamruzzaman, Mahmudur Rahman, Mohammad Lutfar Rahman, Li Su, Yu-mei Hsueh, Marc Weisskopf, Brent Coull, David Chistopher Christiani

**Affiliations:** 1 Department of Environmental Health, Harvard T.H. Chan School of Public Health, 677 Huntington Ave, Boston, MA 02115 USA; 2 Dhaka Community Hospital Trust, 190 Wireless Railgate, 1 Baro Moghbazar, Dhaka, Bangladesh; 3 Harvard Medical School, Department of Population Medicine, Harvard Pilgrim Health Care Institute, 401 Park Drive, Suite 401, Boston, MA 02215 USA; 4 Department of Family Medicine, Shuang Ho Hospital, Taipei Medical University, Taipei City, Taiwan; 5 Department of Public Health, School of Medicine, College of Medicine, Taipei Medical University, No. 250, Wuxing Street, Xinyi District, Taipei City, Taiwan; 6 Department of Epidemiology, Department of Environmental Health, Harvard T.H. Chan School of Public Health, 677 Huntington Ave, Boston, MA 02115 USA; 7 Department of Biostatistics, Department of Environmental Health, Harvard T.H. Chan School of Public Health, 677 Huntington Ave, Boston, MA 02115 USA; 8 Pulmonary and Critical Care Unit, Department of Medicine, Massachusetts General Hospital, 55 Fruit St, Boston, MA 02114 USA

**Keywords:** Environmental arsenic exposure, Arsenic metabolism, Urinary arsenic metabolites, Pregnancy, Reproductive health

## Abstract

**Background:**

Prenatal inorganic arsenic (iAs) exposure is associated with pregnancy outcomes. Maternal capabilities of arsenic biotransformation and elimination may influence the susceptibility of arsenic toxicity. Therefore, we examined the determinants of arsenic metabolism of pregnant women in Bangladesh who are exposed to high levels of arsenic.

**Methods:**

In a prospective birth cohort, we followed 1613 pregnant women in Bangladesh and collected urine samples at two prenatal visits: one at 4–16 weeks, and the second at 21–37 weeks of pregnancy. We measured major arsenic species in urine, including iAs (iAs%) and methylated forms. The proportions of each species over the sum of all arsenic species were used as biomarkers of arsenic methylation efficiency. We examined the difference in arsenic methylation using a paired t-test between first and second visits. Using linear regression, we examined determinants of arsenic metabolism, including age, BMI at enrollment, education, financial provider income, arsenic exposure level, and dietary folate and protein intake, adjusted for daily energy intake.

**Results:**

Comparing visit 2 to visit 1, iAs% decreased 1.1% (*p* <  0.01), and creatinine-adjusted urinary arsenic level (U-As) increased 21% (95% CI: 15, 26%; *p* <  0.01). Drinking water arsenic concentration was positively associated with iAs% at both visits. When restricted to participants with higher adjusted urinary arsenic levels (adjusted U-As > 50 μg/g-creatinine) gestational age at measurement was strongly associated with DMA% (β = 0.38, p <  0.01) only at visit 1. Additionally, DMA% was negatively associated with daily protein intake (β = − 0.02, p <  0.01) at visit 1, adjusting for total energy intake and other covariates.

**Conclusions:**

Our findings indicate that arsenic metabolism and adjusted U-As level increase during pregnancy. We have identified determinants of arsenic methylation efficiency at visit 1.

## Introduction

Inorganic arsenic (iAs) is a ubiquitous, naturally occurring environmental toxicant [1, 2]. It has been linked to cancers [3] as well as increased risk of cardiovascular conditions [4], pregnancy complications [5], and developmental impairments [6]. iAs is a serious public health concern, as 200 million people worldwide are exposed to arsenic levels in drinking water that exceed the World Health Organization’s recommended limit of 10 μg/L [7].

Pregnant women and developing fetuses are especially susceptible to arsenic exposure. Arsenic-related adverse health conditions during pregnancy include anemia, nausea, vomiting, and abnormal cramping [5, 8]. Prenatal arsenic exposure is linked to reduced gestation time, low birth weight, spontaneous abortion, stillbirth, neonatal mortality, and infant mortality [9–17]. Identifying factors that influence susceptibility to arsenic toxicity in mothers and children can provide knowledge for risk assessment and guide effective interventions in underserved arsenic-endemic areas.

The human body metabolizes arsenic primarily in the liver. Ingested arsenate (iAs^V^) is reduced to arsenite (iAs^III^) by arsenate reductase, reacts with glutathione, and is enzymatically methylated to monomethylarsonous acid (MMA^III^) by the methyl-donor S-adenosyl methionine (SAM). MMA^III^ is either rapidly oxidized to monomethylarsonic acid (MMA^V^) or is involved in another cycle of methylation to form dimethylarsinous acid (DMA^III^), which is then oxidized to dimethylarsinic acid (DMA^V^) [18]. Among organic forms of arsenic, trivalent forms (MMA^III^ and DMA^III^) are highly toxic and are reactive intermediates in the arsenic methylation pathway, while pentavalent forms (MMA^V^ and DMA^V^) are less toxic and are more readily excreted in urine [19]. The valence of organic arsenics are difficult to detect in human urine [20], but the proportions of urinary excretion of iAs, monomethyl forms of arsenic (MMA), and dimethyl forms of arsenic (DMA) are often used to evaluate arsenic methylation in vivo [21].

Maternal arsenic methylation efficiency is an important modifier of arsenic-related negative pregnancy outcomes. A lower proportion of DMA and a higher proportion of iAs may indicate decreased arsenic methylation, leading to increased iAs retention [22–25]. Epidemiological studies show that arsenic methylation efficiency can modify arsenic-related health risks in exposed pregnant women, as a greater proportion of iAs in urine is associated with impaired fetal growth [26, 27].

Many factors influence methylation of arsenic species, including arsenic exposure level, age, body mass index (BMI), sex, smoking, genetic factors, ethnicity, pregnancy, breastfeeding [28, 29], and genetic factors [30]. In addition, growing evidence suggest that nutrition influences arsenic metabolism and the risk of arsenic-related morbidity. Pregnant women undergo complex hormonal changes, which influence a remarkable increase in arsenic methylation during pregnancy [31–33]. Patterns and determinants of arsenic metabolism need to be studied separately in pregnant women not only because of their changed metabolism, but also because developing children are especially vulnerable to external toxicants [32, 34].

Arsenic exposure from drinking water is most severe in Bangladesh, where it is estimated that > 19 million people are exposed to > 5 times the standard arsenic level [35]. Therefore, we studied arsenic methylation and excretion profiles during pregnancy in a Bangladeshi cohort, as well as factors that may influence arsenic metabolism, including gestational weeks, water arsenic exposure, socioeconomic variables, dietary folate intake, and dietary protein intake. Together, these observations seek to provide a better understanding of how pregnancy affects arsenic metabolism.

## Methods

### Study population

During 2008–2011, we recruited 1613 pregnant women from two study sites in Bangladesh: one located in Sirajdikhan, a suburban upazila (subdistrict) located 29 km south of the capital city Dhaka; and the other in Pabna, a rural upazila located 122 km northwest from Dhaka [5, 36]. Eligible participants were adult women (≥18 years old) in their first trimester of pregnancy with a single fetus and who had a complete drinking water history that included tube well usage up to 6 months before pregnancy. These women participated in Dhaka Community Hospital’s (DCH) prenatal health program and planned to deliver at home with a DCH-trained midwife or at a DCH hospital or clinic. All participating families signed the informed consent of the study.

At enrollment at weeks 4–16 of pregnancy (visit 1), trained DCH healthcare workers who lived in the local area administered questionnaires to collect information on participants’ demographics, lifestyle, and medical condition. We collected a drinking water sample to assess arsenic exposure level, a urine sample to assess arsenic metabolite profile, and a toenail sample from each participant to assess long-term arsenic exposure levels. In addition, we provided all participants with a daily prenatal multivitamin with folate (400 μg) upon recruitment. Due to ethical responsibility, we advised participants to avoid using contaminated water sources.

The follow-up visit (visit 2) occurred at 21–37 weeks of pregnancy. Healthcare workers collected another urine sample and administered a locally validated food frequency questionnaire (FFQ) to acquire habitual dietary intake information from the previous 12 months [37]. At the time of visit 2, 1438 participants remained in the study. Reasons for loss-of-follow-up included loss of contact (*N* = 6), participant dropout (*N* = 18), miscarriage (*N* = 131), stillbirth (*N* = 11), sample failure (*N* = 2), missing samples (*N* = 2), and twin pregnancy (*N* = 5). After visit 2, we followed participants until one-month post-partum and collected their drinking water samples and toenail samples again to assess the stability of arsenic exposure.

### Urinary arsenic metabolite concentration

For each participant, we collected spot urine samples at both visits 1 and 2 to assess concentrations of arsenic methylation species, including iAs^III^, iAs^V^, MMA, and DMA. At the time of each visit, healthcare workers provided each participant a urine cup and instructed them how to obtain enough urine for analysis. Urine samples were sealed appropriately, brought to the local laboratory in iceboxes, and stored in a − 20 °C freezer. Frozen urine samples were shipped to the Department of Public Health, School of Medicine at Taipei Medical University, Taiwan, where they were stored in − 80 °C freezers. At the time of analysis, the urine samples were thawed at room temperature, sonicated for dispersion, filtered through a Sep-Pak C18 column (Mallinckrodt Baker Inc., NJ, USA), and transferred into 200-μL aliquots. We separated arsenic species fractions by high-performance liquid chromatography (Waters 501, Waters Associates, Milford, MA, USA) with columns from Phenomenex (Nucleosil, Torrance, CA, USA). Concentrations of the 4 urinary arsenic metabolites were determined by hydride generator atomic absorption spectrometry (PerkinElmer, Waltham, MA, USA). Standard reference material no. 2670a was obtained from National Institute of Standards and Technology (Gaithersburg, MD, USA), and recovery rates for iAs^III^, iAs^V^, MMA, and DMA ranged 93.8–102.2%. The limit of detection (LOD) for each urinary arsenic metabolite was three times the standard deviation of 10 runs: 0.02 μg/L, 0.06 μg/L, 0.07 μg/L, and 0.10 μg/L for iAs^III^, iAs^V^, MMA, and DMA, respectively [38]. Numbers of observations under the LOD were 810 (50%), 476 (30%), 316 (20%), and 1 (0%) for visit 1; and 841(58%), 374 (26%), 172 (12%), and 1 (0%) for visit 2, for iAs^III^, iAs^V^, MMA, and DMA, respectively. We kept records below the LOD at their original value in our statistical analyses to provide the most information [39, 40]. To control for urine dilution, we analyzed the concentration of urinary creatinine using a colorimetric assay (Modular P800, Roche Inc., Mannheim, Germany).

### Drinking water arsenic concentration

To assess drinking water arsenic exposure level (DW-As) and its stability during the follow-up period, two repeated drinking water samples were collected as previously described [5] at visit 1 and one-month post-partum. Briefly, tube well water samples were collected from the participant’s primary water source using well pumps after a one-minute purge. Samples were transferred to acid-washed polyethylene bottles and acidified with nitric acid for storage. Samples were then shipped to the laboratory (Environmental Laboratory Services, North Syracuse, NY, USA) and analyzed using a hybrid-generation technique of high-resolution inductively coupled process spectrometry (ICP-MS), following US Environmental Protection Agency method 200.8. Machines were sensitive to 1 μg/L of total arsenic concentration. Records with < 1 μg/L (*N* = 329, 20.5%) were assigned a value of 0.5 μg/L for statistical analyses (Kile et al. 2014).

### Toenail samples

To assess long-term arsenic levels in the body, we collected and analyzed maternal toenail samples at visit 1 and at one-month post-partum as previously described [9, 41]. Briefly, toenail samples were digested with Optima nitric acid (Thermo Fisher Scientific, Waltham, MA, USA) and analyzed using ICP-MS for total toenail arsenic concentration (T-As) [42].

### Dietary assessment

To obtain recalled food consumption frequency during the previous 12 months, we administered a locally-validated semi-quantitative FFQ at visit 2 [37]. We included 42 common food items in Bangladesh from five categories: [1] cereal and bread [2]; vegetables [3]; legumes, pulses, and seeds [4]; fish, poultry, meat, and eggs; and [5] milk-based food items. The scale of consumption frequency and portion sizes were as previously described [37]. Trained technicians entered FFQ data. Daily protein intake (g/day), folate intake (μg /day), and energy intake (kcal/day) were estimated using the 2013 Food Composition Table for Bangladesh [43]. For dish types that were not available in the questionnaire table, nutrient compositions were calculated based on average weighted recipes provided by local dietitians at DCH using nutrient retention factors and yield factors in the Food Composition Table.

### Statistical analysis

We adjusted urinary arsenic metabolite concentrations (mg/g-creatinine) by absolute concentration (μg/L) divided by urinary creatinine concentration (mg/dL). Adjusted total urinary arsenic was the sum of adjusted urinary arsenic metabolites. The proportion of each urinary arsenic metabolite (iAs^III^%, iAs^V^%, MMA%, and DMA%) was calculated using its concentration divided by the sum of all urinary arsenic metabolite concentrations. The proportion of iAs (iAs%) was the sum of iAs^III^% and iAs^V^%.

We used Wilcoxon signed-rank test to compare the percentages of arsenic species in urine between two visits and paired t-tests to compare the log-transformed concentration of arsenic species, creatinine levels, and total urinary arsenic levels. We calculated Spearman correlations of urinary arsenic metabolites.

Linear regression models were used to examine potential determinants of arsenic metabolism biomarkers. iAs%, MMA%, and DMA% were treated as separate outcome variables. Independent variables were age, BMI at enrollment, arsenic exposure level (adjusted U-As, continuous), education level (<secondary education or ≥ secondary education), monthly income of the financial provider, daily protein intake, daily folate intake, and daily energy intake tertiles (low/medium/high). We fitted each visit and study site to a separate model. Based on previous literature, variables that are likely to be correlated with exposure and outcome were considered in models. Analysis was applied to all mothers, as well as to a restricted sample of participants with adjusted U-As of > 50 μg/g creatinine. We chose a cut-off point of 50 μg/g-creatinine because this level is around the 25th quantile of exposure (see Additional file 1: Table S1). Further, we excluded some outlying measurements from analysis (exclusion criteria: creatinine level < 10 mg/dL and adjusted U-As > 3000 μg/g-creatinine).

We separately compared the whole study population (*N* = 1613) and participants with no missing data (*N* = 1425 at visit 1; *N* = 1420 at visit 2). There were no significant differences in demographics or exposure level between the two study populations. All statistical analyses were performed with SAS software (version 9.4; SAS Institute Inc., Cary, NC, USA).

## Results

Most participants were Bangladeshi homemakers (99%), non-smokers (100%), did not chew tobacco or betel nut (99%), and reported physical activity during pregnancy as “on their feet all day but in a stationary position or only spend half the day moving around on their feet” (99%). Detailed information about participants’ demographics, lifestyle, and exposure information is shown in Table 1. Participants from Pabna had a higher average DW-As exposure level (79.5 ± 131.3 μg/L) compared to participants from Sirajdikhan (12.2 ± 47.4 μg/L). All participants reported taking prenatal vitamins with folate every day from visit 1 to visit 2. There were no significant differences in demographics or exposure level between the sample of all enrolled mothers and completed cases.

Medians and 5th–95th percentiles of the proportions and concentrations of urinary arsenic metabolites at visit 1 and visit 2 are summarized in Table 2. Results of Wilcoxon signed-rank tests and paired t-tests of difference in each arsenic metabolite between the two visits are shown in Table 3. Median iAs% for all mothers decreased from 8.5% at visit 1 to 6.6% at visit 2 (mean change = − 1.1%; *p* <  0.01), mainly due to decreased iAs^III^% (mean change = − 1.6%; *p* <  0.01). Median DMA% for all mothers increased from 85.7% at visit 1 to 87.9% at visit 2 (mean change = 1.1%; *p* <  0.01). No change was observed in MMA% for all mothers.

**Table 1:** Characteristics of study participants in Sirajdikhan and Pabna upazilas, Bangladesh

	Sirajdikhan (*N* = 879)	Pabna (*N* = 727)	All mothers (*N* = 1176)
Years of age, visit 1^a^			
	22.8 ± 4.1	23.0 ± 4.2	22.9 ± 4.2
Education level^b^			
< Secondary education	446 (51%)	335 (46%)	781 (49%)
≥ Secondary education	431 (49%)	389 (54%)	820 (51%)
BMI, visit 1 (kg/m^2^)			
	21.0 ± 3.3	20.0 ± 2.9	21.0 ± 3.0
Financial provider’s monthly income (taka)			
Unknown	9 (1%)	23 (3%)	32 (2%)
0–2000	5 (1%)	10 (1%)	15 (1%)
2001–3000	39 (4%)	197 (27%)	236 (15%)
3001–4000	178 (20%)	222 (31%)	400 (25%)
4001–5000	328 (37%)	167 (23%)	495 (31%)
5000–6000	190 (22%)	63 (9%)	253 (16%)
> 6000	129 (15%)	44 (6%)	173 (11%)
Gestational weeks, visit 1			
	11.1 ± 3.1	11.4 ± 3.0	11 ± 3.0
Gestational weeks, visit 2			
	28.6 ± 1.8	29.3 ± 1.9	28.9 ± 1.9
Environmental smoke exposure			
No	555 (63%)	370 (51%)	925 (58%)
Yes	322 (37%)	356 (49%)	678 (42%)
Number of glasses of water drank per day			
	7.7 ± 2.3	7.7 ± 2.4	8 ± 2.0
Drinking water arsenic exposure categories			
Quartile 1: ≤0.89 μg/L	309 (35%)	92 (13%)	401 (25%)
Quartile 2: 0.89–2 μg/L	397 (45%)	24 (3%)	421 (26%)
Quartile 3: 2–33 μg/L	103 (12%)	278 (38%)	381 (24%)
Quartile 4: > 33 μg/L	69 (8%)	332 (46%)	401 (25%)
Drinking water arsenic (μg/L), visit 1			
	12.1 ± 47.4	79.5 ± 131	42.6 ± 101
Median	1.4	27.0	2.0
Drinking water arsenic (μg/L), one-month post-partum^c^			
	6.8 ± 32.5	78.9 ± 133	42.7 ± 103
Median	1.0	26.0	1.8
Maternal toenail arsenic (μg/g), visit 1			
	2.2 ± 3.2	4.8 ± 5.9	3.4 ± 4.9
Median	1.1	2.5	1.7
Maternal toenail arsenic (μg/g), one-month post-partum^d^			
	1.8 ± 3.6	3.6 ± 4.2	2.7 ± 4.0
Median	0.7	2.0	1.2
Daily dietary folate intake (μg)			
	268 ± 96.7	396 ± 103	327 ± 118
Daily dietary protein intake (g)			
	131 ± 47.9	219.3 ± 60.0	172 ± 69.6
Daily energy intake (kcal)			
	3195 ± 978	3234 ± 763	3214 ± 885

^a^ Continuous variables are presented as mean ± standard deviation

^b^ Categorical variables are presented as number of participants (percentage)

^c^ Pearson’s correlation of drinking water arsenic concentration between the first visit and one-month post-partum is 0.72 (*p* < 0.0001)

^d^ Pearson’s correlation of toenail arsenic concentration between the first visit and one-month post-partum is 0.84

**Table 2:** Urinary arsenic metabolites of study participants in Sirajdikhan and Pabna at visits 1 and 2

	Sirajdikhan		Pabna		All mothers	
	Visit 1	Visit 2	Visit 1	Visit 2	Visit 1	Visit 2
N	879	783	727	662	1606	1445
iAs^III^%^a^	0 (0, 12.6)	0 (0, 9.9)	3.1 (0, 17.8)	0 (0, 11.5)	0 (0, 15.9)	0 (0, 10.6)
iAs^V^%	1.1 (0, 20.9)	1.8 (0, 26.2)	3.8 (0, 18.6)	4 (0, 20)	2.1 (0, 19.6)	2.6 (0, 23.6)
iAs%	5.7 (0, 24.1)	5.3 (0, 27.7)	11.2 (1.4, 24)	8 (0, 22.1)	8.5 (0, 24)	6.6 (0, 25.4)
MMA%	3.5 (0, 13.4)	4.3 (0, 11.8)	6 (0.6, 13.1)	5.6 (0.1, 11.3)	4.9 (0, 13.2)	4.8 (0, 11.6)
DMA%	89.5 (66.9, 100)	89.6 (66, 99.9)	82 (66.2, 95.6)	85.8 (68.9, 98)	85.7 (66.6, 100)	87.9 (67.3, 99.5)
iAs^III^, μg/L	0 (0, 8.8)	0 (0, 7.7)	1.7 (0, 57.9)	0 (0, 27.8)	0 (0, 28.9)	0 (0, 16.7)
iAs^V^, μg/L	0.2 (0, 8.8)	0.5 (0, 12)	2.5 (0, 36)	2.1 (0, 51.9)	0.6 (0, 21.8)	0.9 (0, 22.5)
iAs, μg/L	0.7 (0, 17.8)	1.1 (0, 17.9)	7.5 (0.2, 81.8)	4.9 (0, 75.9)	2.5 (0, 52.2)	2.4 (0, 39.1)
MMA, μg/L	0.5 (0, 10.6)	1.1 (0, 11)	4.1 (0.1, 42.5)	3.4 (0, 35.1)	1.3 (0, 30.3)	1.7 (0, 21.5)
DMA, μg/L	10.9 (1.9, 121)	20.5 (3.6, 128)	54.2 (8.8, 431)	50.2 (7.4, 371)	22.5 (2.6, 297)	30 (4.5, 259)
U-As	12.2 (2, 147)	23.7 (4.3, 151)	69.9 (9.7, 531)	63.2 (8, 480)	26.5 (2.9, 376)	35.2 (5.1, 325)
U-creatinine, mg/dL	23.1 (6, 113)	34.6 (8.7, 126)	41.4 (11.6, 181)	35 (9.7, 117)	30.3 (7.5, 159)	34.9 (9.1, 123)
Adjusted iAs^III b^	0 (0, 14.9)	0 (0, 10.9)	3.5 (0, 67.6)	0 (0, 50.5)	0 (0, 48.3)	0 (0, 27.7)
Adjusted iAs^V^	0.6 (0, 33.4)	1.3 (0, 36.5)	5.5 (0, 98.1)	6.1 (0, 123)	1.8 (0, 67.5)	2.5 (0, 77.4)
Adjusted iAs	2.7 (0, 51.6)	3.1 (0, 48.9)	15.2 (0.8, 142)	12.6 (0, 156)	6.5 (0, 98.9)	5.7 (0, 104)
Adjusted MMA	1.7 (0, 36)	2.6 (0, 24.4)	8.2 (0.4, 76.6)	8.3 (0.1, 72.5)	3.8 (0, 57.9)	4.3 (0, 52.5)
Adjusted DMA	43.2 (13.8, 369)	52.8 (21.2, 276)	114 (30.1, 577)	126 (39, 776)	65.1 (17.5, 530)	76.4 (24.8, 605)
Adjusted U-As	48.6 (16.2, 470)	61.6 (24.6, 338)	139 (37.4, 784)	154 (43.6, 972)	77.5 (19.6, 677)	90.4 (28.8, 770)

^a^ Proportions of urinary arsenic metabolites are presented as median and 5th–95th percentiles

^b^ Concentrations of urinary arsenic metabolites are creatinine-adjusted (μg/g-creatinine) and presented as median and 5th–95th percentiles

**Fig. 1:**
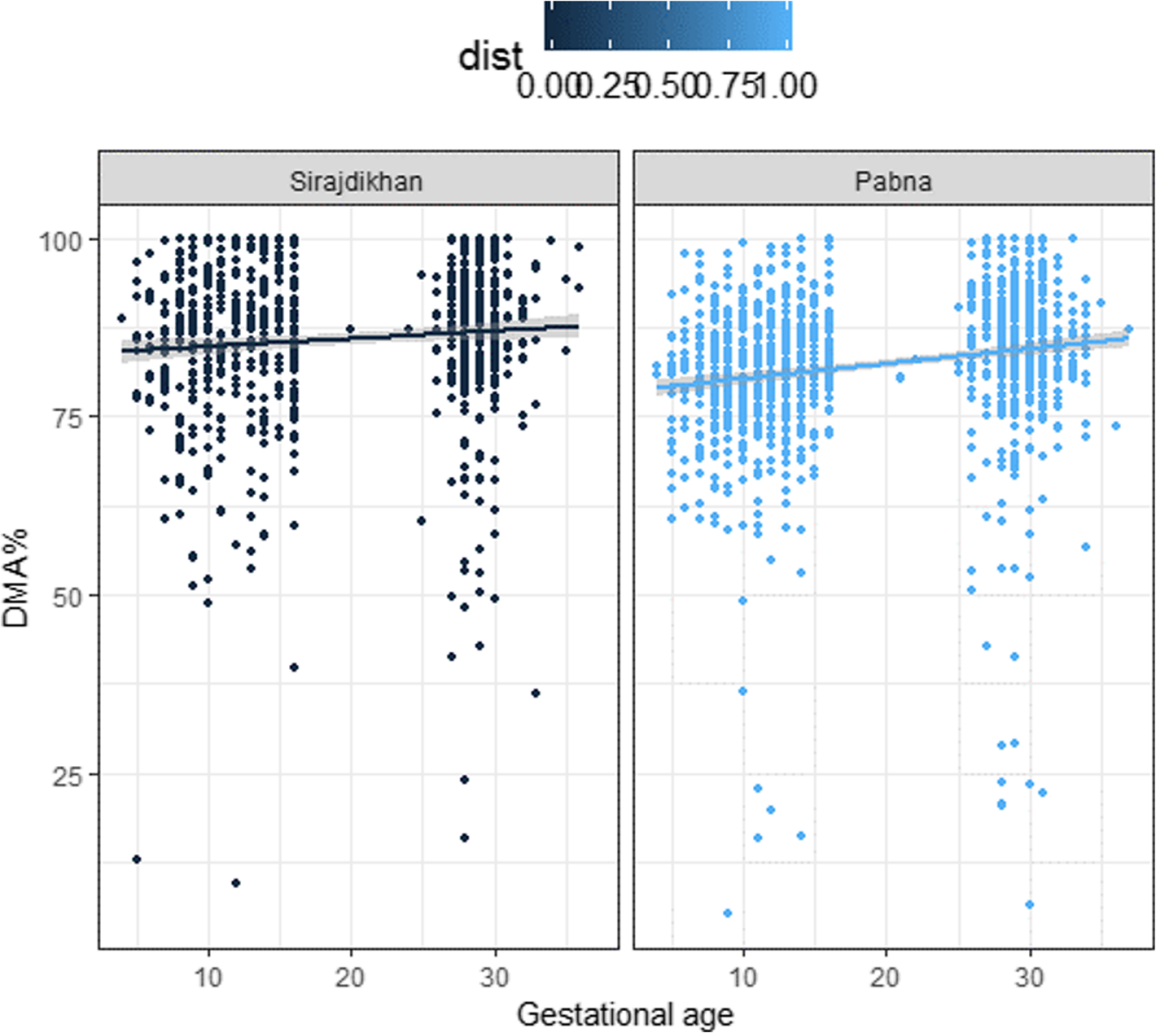
Scatter plots of DMA% and gestational weeks, restricted to participants with adjusted U-As > 50 μg/g-creatinine. [Figure legend: DMA% (%) plotted over gestational age (weeks), showing arsenic methylation efficiency of each participant at two repeated measurements. We applied a linear regression method to fit a solid line with the shade of standard error. We restricted exposure level to adjusted U-As > 50 μg/g-creatinine, which is ~25th percentile of adjusted U-As of both visits 1 and 2. Weeks of gestation was determined by ultrasound examination performed by trained healthcare workers. Dark blue dots indicate observations in Sirajdikhan; light blue dots indicate observations in Pabna]

**Fig. 2:**
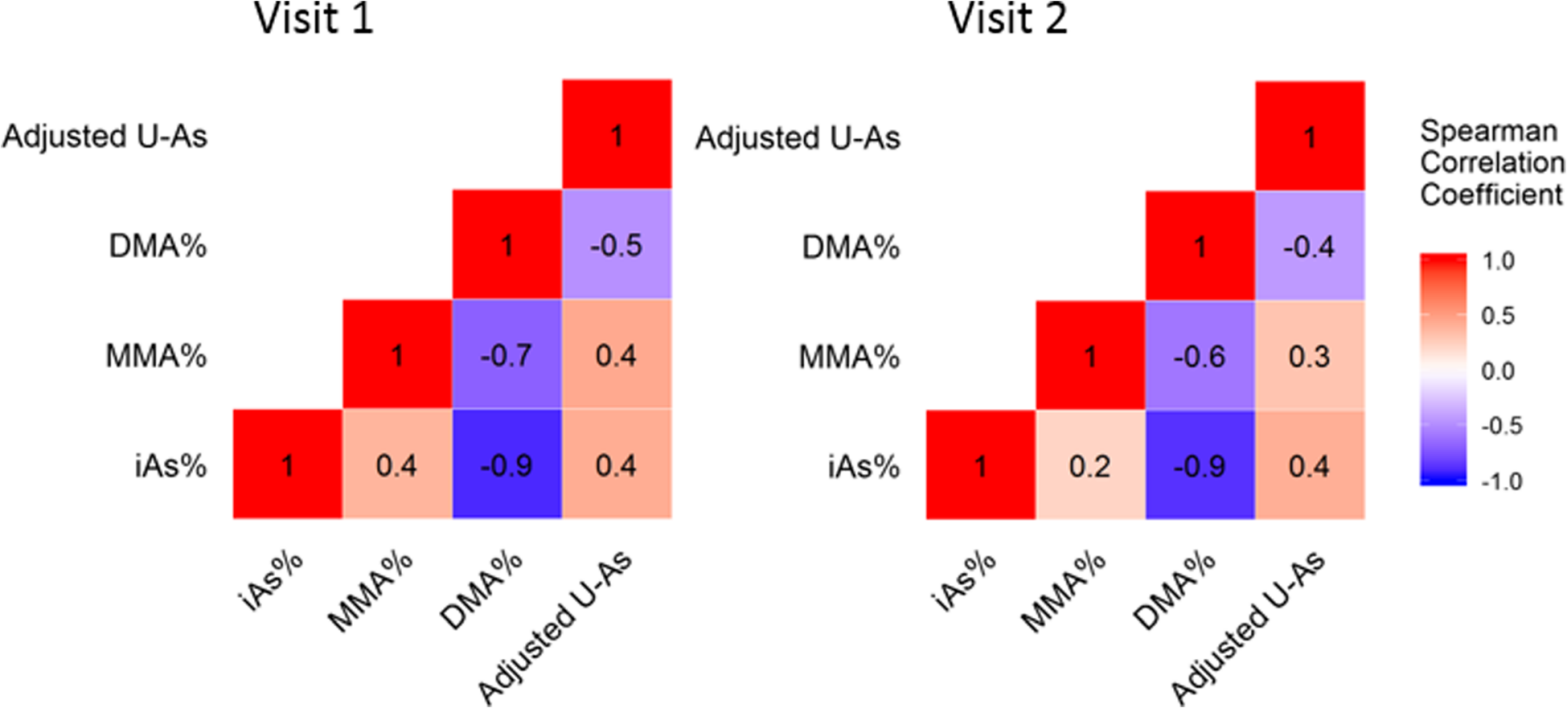
Spearman correlation coefficients for arsenic metabolites at visit 1 and 2. [Figure legend: The unit of adjusted U-As is μg/g-creatinine, and the unit of iAs%, MMA% and DMA% are all “%”. All Spearman correlation coefficients had *p* < 0.01. The number of participants at visit 1 is 1605, while the number of participants at visit 2 is 1443]

Restricting analysis to only participants with adjusted U-As of > 50 μg/g-creatinine, DMA% increased during the first trimester and remained high in the mid-to-late gestational period for participants in both Sirajdikhan and Pabna (Fig. 1). The significance of the increase would be shown with later regression analysis results. Data from all mothers are shown in Additional file 1: Figure S1 Further, proportions of arsenic metabolites in urine were highly correlated at visit 1 and visit 2 (Fig. 2). The adjusted U-As were correlated between visit 1 and visit 2 (see Additional file 1: Figure S2). Total urinary arsenic excretion was associated with urinary creatinine excretion (see Additional file 1: Figure S3). Thus, we used adjusted U-As in continuing analyses. Median U-As increased from 77.6 mg/g-creatinine at visit 1 to 90.4 mg/g-creatinine at visit 2. The mean difference of log-transformed U-As from visit 1 to visit 2 was 0.2 (*p* < 0.01), and the average ratio of U-As of visit 2 over visit 1 was 1.2. Log-transformed DW-As from visit 1 to one-month post-partum remained unchanged (*p* = 0.06; average ratio = 1.08). Log-transformed T-As decreased from visit 1 to one-month post-partum, with an average ratio of 0.70 (*p* < 0.01).

**Fig. 3:**
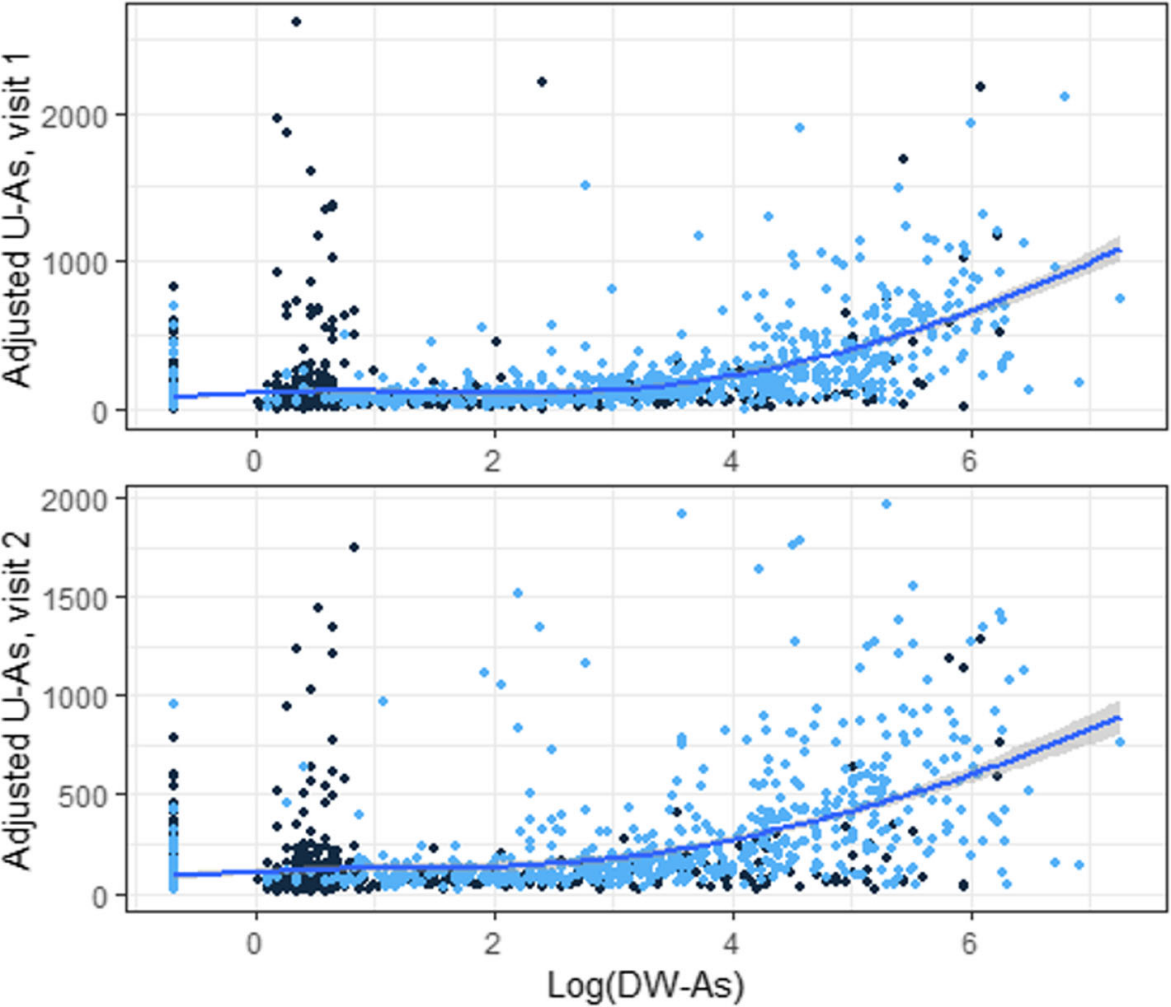
Scatter plot of adjusted U-As over log-transformed DW-As. [Figure legend: Adjusted U-As (unit of adjusted U-As: μg/g-creatinine) was plotted over log-transformed DW-As (unit of DW-As: μg/L), showing the correlation between environmental arsenic exposure and arsenic excretion. We applied Loess local polynomial linear regression to fit a solid curve line with the shade of standard error. Dark blue dots indicate observations in Sirajdikhan; light blue dots indicate observations in Pabna]

The association between adjusted U-As and DW-As is shown in Fig. 3. Because participants in Sirajdikhan had high U-As levels despite low DW-AS levels, they seemed to have other sources of arsenic exposure in addition to drinking water. We applied a linear model for dose-response relationship using data in Pabna, because participants in Pabna were less likely to be affected by other arsenic sources, resulting in a relationship of log (adjusted U-As) = 4.06 + 0.32 × log (DW-As). Table 4 shows determinants affecting the proportions of urinary arsenic metabolites at visit 1. When restricted to participants with adjusted U-As of > 50 μg/g-creatinine, gestational age at measurement was strongly associated with DMA% (β = 0.38, *p* < 0.01). Maternal age was positively associated with DMA% (β = 0.15, *p* = 0.04). Maternal higher education level was negatively associated with iAs% (β = − 1.16, *p* = 0.03). Additionally, DMA% was negatively associated with daily protein intake (β = − 0.02, *p* < 0.01). Every 100 g of daily dietary protein was associated with a 2% decrease of DMA in participants’ urine, adjusting for total energy intake and other covariates. Average iAs% for low, medium, and high tertiles of protein intake was 8.8, 9.3, and 11.8%, respectively. Average DMA% for low, medium, and high tertiles of protein intake was 85.2, 84.3, and 84.9%, respectively.

**Table 3:** Change in percentages of urinary arsenic metabolites and other arsenic biomarkers between two visits^a^

	Sirajdikhan			Pabna			All mothers		
	Median change	Mean	*p*-value^a^	Median change	Mean	*p*-value	Median change	Mean	*p*-value
	(5th–95th percentile)	change		(5th–95th percentile)	change		(5th–95th percentile)	change	
iAs^III^%^b^	0 (−10.6, 8.5)	−0.7	< 0.01	− 0.1 (− 15.7, 9)	−2.6	< 0.01	0 (− 13.9, 8.7)	− 1.6	< 0.01
iAs^V^%	0 (− 13.8, 18.3)	0.8	0.09	0 (−14.2, 13.8)	0.2	0.38	0 (− 14.2, 16.6)	0.5	0.56
iAs%	0 (−16.3, 17.6)	0.1	0.27	−2.7 (−15.9, 12.3)	−2.5	< 0.01	−1.4 (− 16.2, 15.1)	−1.1	< 0.01
MMA%	0.4 (−9.1, 9)	0.5	< 0.01	−0.3 (−8, 5.7)	− 0.6	< 0.01	0.1 (− 8.6, 7.3)	0	0.91
DMA%	−0.2 (−22.7, 21)	− 0.6	0.52	3.3 (−12.2, 20.6)	3.1	< 0.01	1.8 (−19.7, 20.8)	1.1	< 0.01
log (unadjusted U-As)^c^	0.6 (−1.8, 2.8)	0.5	< 0.01	−0.1 (−2.2, 2)	− 0.1	0.03	0.3 (−2, 2.5)	0.2	< 0.01
log(U-creatinine)	0.3 (−1.7, 2.1)	0.3	< 0.01	−0.2 (−2.1, 1.5)	− 0.3	< 0.01	0.1 (− 1.9, 1.9)	0.1	0.07
log (adjusted U-As)	0.6 (−1.8, 2.8)	0.5	< 0.01	−0.1 (−2.2, 2)	− 0.1	< 0.01	0.3 (− 2, 2.5)	0.2	< 0.01
log (DW-As)^d^	0 (−3.5, 2.5)	0	0.89	0 (−1.3, 2)	0.2	< 0.01	0 (−2.7, 2.5)	0.1	0.05
log(T-As)	−0.4 (−1.8, 1.1)	− 0.4	< 0.01	− 0.3 (− 1.1, 0.7)	−0.3	< 0.01	− 0.4 (− 1.5, 0.8)	−0.4	< 0.01

^a^ The units of iAs^III^%, iAs^V^%, iAs%, MMA% and DMA% are all “%”. The unit of unadjusted U-As, U-creatinine, adjusted U-As, DW-As and T-As are μg/L, mg/dL, μg/g-creatinine, μg/L and μg/g, respectively

^b^ Wilcoxon signed rank test *p*-values for the first five variables, conditional on Sirajdikhan site (*N* = 782), Pabna site (*N* = 661), and all mothers (*N* = 1443)

^c^ Paired t-tests of differences between visit 1 and visit 2, conditional on Sirajdikhan site (*N* = 782), Pabna site (*N* = 661), and all mothers (*N* = 1443)

^d^ Paired t-tests of differences between visit 1 and one-month post-partum, conditional on Sirajdikhan site (*N* = 577), Pabna site (*N* = 573), and all mothers (*N* = 1150)

**Table 4:** Determinants of the proportion of arsenic metabolites in urine during early gestation (visit 1)

	All mothers (*N* = 1595)						Restricted sample (adjusted U-As > 50 μg/g-creatinine; *N* = 1066)^a^					
	iAs%		MMA%		DMA%		iAs%		MMA%		DMA%	
	β	*p*-value	β	*p*-value	β	*p*-value	β	*p*-value	β	*p*-value	β	*p*-value
Intercept	12.98	< 0.01	9.09	< 0.01	77.92	< 0.01	12.01	< 0.01	6.42	< 0.01	81.57	< 0.01
Gestational age at measurement (week)	−0.13	0.21	−0.26	< 0.01	0.39	< 0.01	−0.17	0.03	−0.21	< 0.01	0.38	< 0.01
Maternal age (year)	−0.17	0.03	−0.02	0.59	0.19	0.03	−0.15	0.02	0	0.95	0.15	0.04
BMI at enrollment (kg/m^2^)	0.01	0.96	−0.1	0.02	0.09	0.41	−0.02	0.85	−0.06	0.11	0.07	0.43
Education level (≥secondary education)	−0.83	0.20	0.20	0.45	0.63	0.37	−1.16	0.03	0.36	0.12	0.80	0.17
Income of financial provider (≥3000 taka)	−0.68	0.30	0.52	0.05	0.16	0.82	−0.68	0.20	0.07	0.76	0.61	0.31
Adjusted U-As (μg/g-creatinine)	0.01	< 0.01	0.01	< 0.01	−0.01	< 0.01	0.01	< 0.01	0	< 0.01	−0.01	< 0.01
Daily protein intake (g)	0.02	0.01	0	0.67	−0.02	0.01	0.02	< 0.01	0	0.47	−0.02	< 0.01
Daily energy intake (kcal), medium tertile	0.14	0.86	−0.26	0.43	0.12	0.89	−0.45	0.49	0	0.99	0.45	0.54
Daily energy intake (kcal), high tertile	−2.30	0.02	−0.39	0.32	2.69	0.01	−2.53	< 0.01	−0.18	0.59	2.71	< 0.01
Daily folate intake (μg)	0	0.75	0	0.07	0	0.71	0	0.56	0	0.06	−0.01	0.21

^a^ Linear models applied to all mothers, as well as to a restricted sample of participants with adjusted U-As > 50 μg/g creatinine. We selected this cut-off point because it is around the 25th percentile of the distribution of adjusted U-As at both visits. We did not include outliers of adjusted U-As in the analysis, which was > 3000 μg/g-creatinine because of very low creatinine level (< 10 mg/dL) (N = 11)

At visit 2, DMA% was only associated with adjusted U-As. We did not find other determinants of arsenic methylation efficiency at visit 2 (see Additional file 1: Table S2).

## Discussion

Our findings suggest that arsenic methylation efficiency increases during pregnancy. At both visits, higher doses of arsenic were associated with increased iAs% and MMA%, as well as decreased DMA%, which is consistent with previous studies [44–46]. Further, other determinants affected arsenic methylation efficiency only in early gestational periods.

Our study included two study sites in Bangladesh, which were chosen for two reasons: they are under the service area of our collaborator, and they have different exposure levels of arsenic. Sirajdikhan has lower arsenic exposure levels, while Pabna has higher arsenic exposure levels. There were also differences in culture and dietary patterns between the two sites. A group of participants in Sirajdikhan had high levels of adjusted U-As despite low DW-As levels, indicating that these individuals were likely exposed to arsenic from their diets. For example, arsenic can accumulated in rice and rice is one of the common food of Bangladeshi in a daily basis [47, 48]. Participants may also exposed to organic forms of arsenic from sea foods [49]. The exposure misclassification may bias water arsenic’s true association between birth outcomes and developmental health outcomes. Spot urine arsenic level has limited ability to indicate long-term exposure, although it can be a good indicator of exposure from all sources including water.

Arsenic metabolism efficiency usually increases during pregnancy, particularly in the first trimester [31, 32]. Pregnant women usually have an ability to metabolize arsenic and excrete 70–100% of it as DMA in their urine, compared to an average of 60–80% for men and non-pregnant women [29]. We saw the same pattern in our cohort, especially when we restricted analysis to participants with higher arsenic exposure levels. DMA% increased during the first trimester and remained high in the mid-to-late gestational period in our cohort. From our separate linear regression analyses by study visits, arsenic methylation efficiency was associated with gestational age at visit 1 but not at visit 2. The physiological mechanism of these changes was not fully elucidated yet. Reduced iAs^III^% possibly contributed to the decreased iAs%. However, as urine samples were not frozen immediately after collection, it is likely that some iAs^III^ was oxidized to iAs^V^. Thus, we focused our analysis on iAs%, which is not affected by oxidation or reduction.

Other than DMA%, U-As increased at follow-up visits, while DW-As remained unchanged. Toenail arsenic levels can represent the long-term internal dose of iAs over the past 3–6 months [19, 41]. Thus, the two measurements taken at visit 1 and one-month post-partum demonstrate relative arsenic absorption in the time periods before visit 1 through mid-to-late gestation. Our observation of decreased T-As may indicate increased arsenic clearance during pregnancy, although additional evidence is needed to confirm an increase in arsenic excretion. Previous studies hypothesize that increased U-As is partially due to increased DMA%, as DMAs are more readily excreted in urine [31, 32]. However, after excluding outliers of adjusted U-As, we found a negative association between ∆DMA% (DMA% difference between two visits) and ∆U-As (U-As difference between two visits) in linear regression (β = − 6.7, *p* < 0.01), adjusted for age, BMI, education, income, and DW-As. The underlying reason is unclear. It indicates that the improvements of arsenic methylation efficiency may not results in higher arsenic excretion.

Folate plays a key role in one-carbon metabolism, which promotes synthesis of the methyl donor SAM [50]. Several epidemiological studies showed that folate supplementation in folate-deficient cohorts were associated with improved arsenic methylation efficiency and arsenic elimination [51–53]. Our data show that average daily folate intake of participants was 327 μg. The majority of participants had insufficient daily folate intake compared to the recommended dose of 400–800 μg. We were not able to compare folate intake level at visit 1 and 2, because the estimate was based on a self-reported FFQ for the past year. We hypothesized that dietary folate intake was positively associated with arsenic methylation efficiency, but we did not find an association at either visit from regression analyses. At visit 1, participants had not yet received folate supplements, so diet was the main source of folate. This non-significant result about folate intake is in agreement with previous findings that folate levels during pregnancy marginally influence arsenic methylation [32, 34, 54]. At visit 2, every participant had been taking folate supplements, so there was less variation in participants’ folate levels and thus a reduced ability to show any effect. The average daily protein intake was 172 g for all mothers, which is much higher than the US daily recommendation of 75–100 g. Our method may overestimate the protein intake, but it represents the comparative protein intake among participants. Our study shows dietary intake of protein was associated with greater iAs% in the first trimester, which indicates reduced methylation efficiency, adding to the inconsistent literature regarding protein intake and arsenic-related effects. Steinmaus et al. investigated a US population and found that people in the lower quartile of protein intake excreted a higher proportion of ingested iAs as MMA and a lower proportion as DMA, compared to the upper quartile of protein intake [54]. Some human studies have found worse arsenic-associated health effects among those consuming lower amounts of meat, eggs, and vegetables [55, 56]. Kurzius-Spencer et al. found that higher protein intake was associated with a decreased proportion of iAs in urine [57]. However, Heck et al. found that higher intake of protein, methionine, and cysteine was associated with 10–15% greater total urinary arsenic excretion, after controlling for total energy intake, body weight, sex, age, tobacco use, and intake of other nutrients [58]. However, these studies did not properly address the complete biological role of protein intake. Inconsistent study results may be due to inaccurate measures or uncontrolled confounders. In addition, pregnant women compared with non-pregnant adults appear to have enhanced arsenic metabolism [34]. Therefore, factors affecting arsenic metabolism in non-pregnant women may not be applicable to our observations for pregnant women.

In addition, the association between DMA% and protein intake may be slightly confounded by some high-protein dishes, as Lin et al. found that toenail arsenic level is positively associated with consumption of fish and meat items [59]. The effect of dietary protein and folate intake may also be confounded by body size, as participants with different body sizes may proportionally consume less or more food. Nonetheless, our multiple linear regression models adjusted for BMI, and we found similar results in our analyses with and without adjusting for total energy intake.

In this study, we used proportions of arsenic metabolites in urine to represent arsenic methylation efficiency, which is commonly used in epidemiological studies. Ratios between arsenic metabolites were not used here, as many pregnant women have very low proportions of iAs and MMA, resulting in extreme values. We also found similar results in sensitivity analysis on adjusted iAs concentrations (μg/g-creatinine) and its determinants. Daily protein intake was positively associated with adjusted iAs concentration (*p* = 0.07), and the high tertile of daily energy intake had lower average iAs concentration *(p* = 0.69), adjusted for total urinary arsenic, age, BMI at enrollment, gestational age, education, and income of the financial provider.

Despite its strengths, our study also has several limitations. For instance, spot urine samples are only useful for reflecting short-term exposure. Although the percentage of arsenic metabolite is remarkably stable over time [21, 60], the time of day at sample collection may affect the observation of MMA% and iAs% [21, 61]. To minimize this confounding effect, healthcare workers in our study collected urine samples at a similar time of a day. Thus, while we did not control for bias from this confounder, it should not significantly bias the associations of arsenic methylation efficiency and its determinants. We observed good association between adjusted U-As and DW-As, indicating that adjusted U-As can be a reliable marker of exposure. Another limitation is that participants’ dietary intake was self-reported and thus subject to reporting bias, especially when we asked about diet over a long period of 12 months. Protein intake was very high compared to the US Dietary Reference Intake for pregnant women [62, 63]. However, FFQs may not accurately capture absolute dietary intake or active dose of nutrients, although FFQs have strong validity in correctly ranking nutrition intake compared to a food diary that does not rely on recall and memory [64]. To mitigate the effects of measurement errors in self-reported FFQs, we controlled for energy intake in regression analyses. Further, although we have food intake amount, we are not able to quantify arsenic in the diet, which could help capture other arsenic sources. Additionally, we only focused on dietary folate and protein intake but lack information on intake of other nutrients related to one-carbon metabolism, including cysteine, methionine, choline, and vitamin B-12 [65]. Future studies should investigate whether these nutrients affect arsenic methylation efficiency during pregnancy and whether nutritional supplementation is beneficial for arsenic detoxification.

This is the first study to evaluate the association between arsenic methylation efficiency and dietary intake of protein and folate in pregnant women. Patterns of arsenic methylation efficiency and total urinary excretion during pregnancy may vary by geographical area, due to variations in demographics, lifestyle, and dietary habits. Therefore, the difference in arsenic methylation efficiency between our two study centers is not fully understood.

## Conclusions

Our findings suggest that both arsenic methylation efficiency and total urinary arsenic level increase during pregnancy in a cohort of women in Bangladesh. The patterns of arsenic methylation efficiency and total arsenic excretion varied by geographic area. Also, we found that greater dietary intake of protein or folate may not improve arsenic methylation efficiency. Future research should continue to study potential approaches to improve health outcomes in populations exposed to arsenic.

## Supplementary information

**Additional file 1: Table S1.** Quantiles of adjusted urinary arsenic concentrations (U-As) (μg/g creatinine).** Table S2.** Determinants of the proportion of arsenic metabolites in urine in mid-to-late gestation (visit 2)**. Figure S1.** Scatter plots of DMA% and gestational weeks for all mothers. **Figure S2.** Correlation between adjusted U-As at visit 1 and visit 2. **Figure S3.** Association between unadjusted U-As and urinary creatinine at visit 1 (top) and visit 2 (bottom).

## Data Availability

The datasets generated and analyzed during the current study are not publicly available due to institutional review board specifications (but are available from the corresponding author on reasonable request).

## Abbreviations

BMI: Body mass index
DCH: Dhaka Community Hospital
DMA: Dimethyl forms of arsenic
DMA%: Proportion of DMA in urine
DMA^III^: Dimethylarsinous acid
DMA^V^: Dimethylarsinic acid
DW-As: Drinking water arsenic level
FFQ: Food frequency questionnaire
iAs: Inorganic arsenic
iAs%: Proportion of iAs in urine
iAs^III^: Arsenite
iAs^V^: Arsenate
ICP-MS: Inductively coupled process spectrometry
MMA: Monomethyl forms of arsenic
MMA%: Proportion of MMA in urine
MMA^III^: Monomethylarsonous acid
MMA^V^: Monomethylarsonic acid
SAM: S-adenosyl methionine
T-As: Toenail arsenic level
U-As: Adjusted total urinary arsenic level
