# Supplementary material for: Determinants of arsenic methylation efficiency and urinary arsenic level in pregnant women in Bangladesh
Source: Environ Health. 2019 Nov 5;18:94. doi: 10.1186/s12940-019-0530-2 (PMC6833186; doi:10.1186/s12940-019-0530-2)

Additional Materials

Table S1 Quantiles of adjusted urinary arsenic concentrations (U-As) (µg/g creatinine)

| **Visit 1** |  | | | | |
| --- | --- | --- | --- | --- | --- |
| Quantiles | 0% | 25% | 50% | 75% | 100% |
| U-As (µg/g creatinine) | 0.4 | 41.5 | 77.2 | 178 | 2604 |
| **Visit 2** |  | | | | |
| Quantiles | 0% | 25% | 50% | 75% | 100% |
| U-As (µg/g creatinine) | 6.9 | 51.4 | 89.8 | 190 | 1964 |

Table S2 Determinants of the proportion of arsenic metabolites in urine in mid-to-late gestation (visit 2)

|  | **All mothers (N = 1595)** | | | | | | **Restricted sample (adjusted U-As >50 µg/g-creatinine; N = 1097)^a^** | | | | | |
| --- | --- | --- | --- | --- | --- | --- | --- | --- | --- | --- | --- | --- |
|  | **iAs%** |  | **MMA%** |  | **DMA%** |  | **iAs%** |  | **MMA%** |  | **DMA%** |  |
|  | **β** | ***p*-value** | **β** | ***p*-value** | **β** | ***p*-value** | **β** | ***p*-value** | **β** | ***p*-value** | **β** | ***p*-value** |
| **Intercept** | 13.08 | 0.01 | 2.04 | 0.30 | 84.88 | <0.01 | 17.54 | <0.01 | 3.65 | 0.05 | 78.81 | <0.01 |
| **Gestational age at measurement (week)** | -0.20 | 0.19 | 0.07 | 0.29 | 0.13 | 0.41 | -0.30 | 0.10 | 0.02 | 0.71 | 0.28 | 0.13 |
| **Maternal age (year)** | -0.07 | 0.34 | 0.02 | 0.59 | 0.05 | 0.49 | -0.05 | 0.55 | 0.02 | 0.43 | 0.03 | 0.74 |
| **BMI at enrollment (kg/m^2^)** | 0.07 | 0.45 | -0.01 | 0.86 | -0.07 | 0.51 | 0.03 | 0.80 | -0.06 | 0.12 | 0.03 | 0.82 |
| **Education level (≥secondary education)** | -0.31 | 0.60 | 0.11 | 0.64 | 0.20 | 0.75 | -0.23 | 0.76 | 0.06 | 0.80 | 0.17 | 0.82 |
| **Income of financial provider (≥3,000 taka)** | -1.42 | 0.02 | 0.26 | 0.29 | 1.16 | 0.07 | -1.51 | 0.04 | 0.78 | <0.01 | 0.73 | 0.33 |
| **Adjusted U-As (μg/g-creatinine)** | 0.01 | <0.01 | 0 | <0.01 | -0.01 | <0.01 | 0.01 | <0.01 | 0 | <0.01 | -0.01 | <0.01 |
| **Daily protein intake (g)** | -0.01 | 0.35 | 0 | 0.45 | 0.01 | 0.55 | -0.01 | 0.16 | 0 | 0.28 | 0.01 | 0.29 |
| **Daily energy intake (kcal), medium tertile** | 0.46 | 0.54 | -0.26 | 0.40 | -0.21 | 0.79 | 1.05 | 0.26 | -0.03 | 0.92 | -1.02 | 0.28 |
| **Daily energy intake (kcal), high tertile** | 0.03 | 0.97 | -0.41 | 0.26 | 0.38 | 0.69 | 0.89 | 0.42 | -0.57 | 0.11 | -0.33 | 0.77 |
| **Daily folate intake (µg)** | 0 | 0.36 | 0 | 0.75 | 0 | 0.32 | 0 | 0.40 | 0 | 0.23 | -0.01 | 0.23 |

^a^ Linear model applied to all mothers, as well as to a restricted sample of participants with adjusted U-As >50 µg/g creatinine.

Fig. S1 Scatter plots of DMA% and gestational weeks for all mothers

DMA% (%) plotted over gestational age (weeks), showing arsenic methylation efficiency of each participant at two repeated measurements. We applied linear regression to fit a solid line with the shade of standard error. Weeks of gestation were determined by ultrasound examination performed by trained healthcare workers. Dark blue dots indicate observations in Sirajdikhan; light blue dots indicate observations in Pabna.


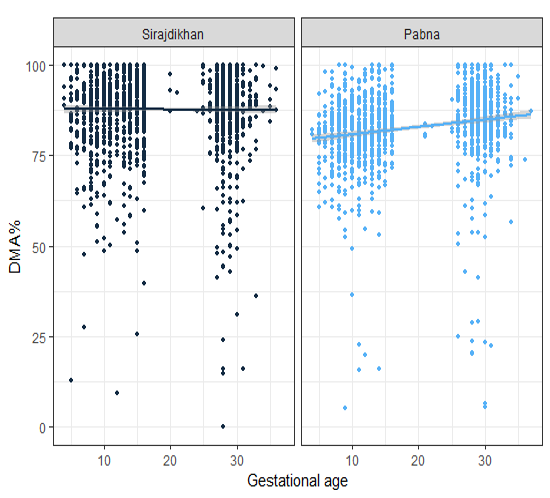


Fig. S2 Correlation between adjusted U-As at visit 1 and visit 2

Log-transformed adjusted U-As (unit of adjusted U-As: μg/g-creatinine) at visit 2 plotted over it at visit 1, showing the correlation of urinary arsenic excretion level between two visits. We applied linear regression to fit a solid line with the shade of standard error.


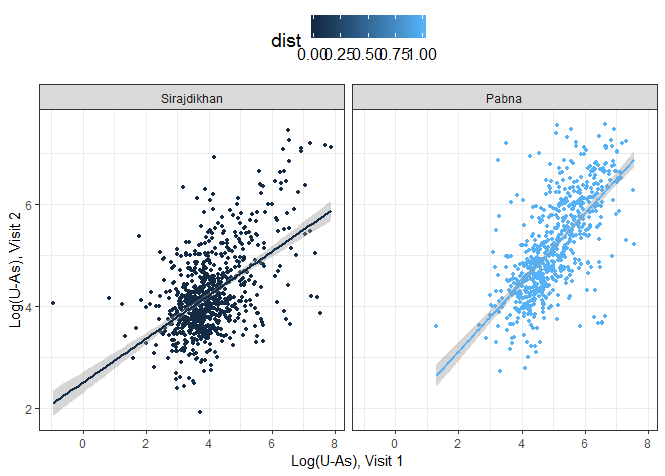


Fig. S3 Association between unadjusted U-As and urinary creatinine at visit 1 (top) and visit 2 (bottom)

Log-transformed adjusted U-As (unit of adjusted U-As: μg/g-creatinine) plotted over log-transformed U-creatinine (unit of U-creatinine: mg/dL), showing the relationship between total urinary arsenic excretion and creatinine level. We applied linear regression to fit a solid line with the shade of standard error. It showed the necessity of creatinine adjustment of urinary arsenic metabolites.


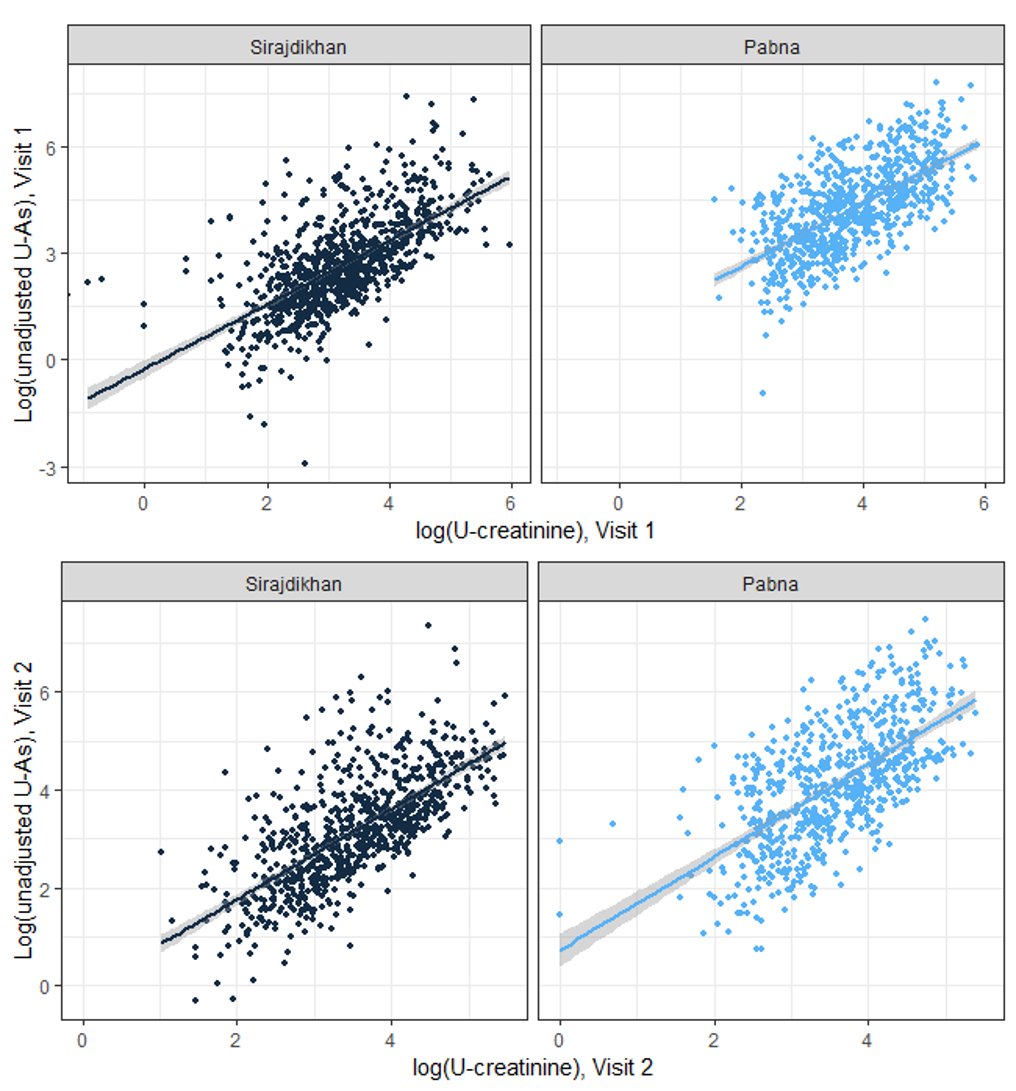

Supplement: Supplementary file 1 — Additional file 1: Table S1. Quantiles of adjusted urinary arsenic concentrations (U-As) (μg/g creatinine). Table S2. Determinants of the proportion of arsenic metabolites in urine in mid-to-late gestation (visit 2). Figure S1. Scatter plots of DMA% and gestational weeks for all mothers. Figure S2. Correlation between adjusted U-As at visit 1 and visit 2. Figure S3. Association between unadjusted U-As and urinary creatinine at visit 1 (top) and visit 2 (bottom). [file 12940_2019_530_MOESM1_ESM.docx]
